# Mobility—A Bridge to Sense of Coherence in Everyday Life: Older Patients’ Experiences of Participation in an Exercise Program During the First 3 Weeks After Hip Fracture Surgery

**DOI:** 10.1177/10497323211008848

**Published:** 2021-04-30

**Authors:** Irene Vestøl, Jonas Debesay, Astrid Bergland

**Affiliations:** 1 Oslo Metropolitan University, Oslo, Norway

**Keywords:** Norway, exercise, older people, rehabilitation, coping, lived experience, health, health promotion, experiences, musculoskeletal disorders, qualitative, semi-structured interviews

## Abstract

Our aim with this article was to explore the experiences of older people who participated in the evidence-based High-Intensity Functional Exercise (HIFE) Program during the first 3 weeks of rehabilitation after hip fracture surgery. Nineteen older people participated in the study. Data were analyzed using systematic text condensation. One overarching theme “Exercise is the key for regaining mobility and a sense of coherence (SOC) in everyday life” emerged from the analysis in addition to these five themes: (a) understanding the existential importance of mobility; (b) maintaining a positive self-image by regaining mobility; (c) regaining one’s old life and independence in everyday living; (d) maintaining interpersonal relationships through mobility; and (e) creating positive emotions by being able to move. The findings highlight the importance of exercise as a strategy for regaining mobility, illustrated by the essential role it played in the participants’ lives after suffering a hip fracture.

## Introduction

Hip fractures represent a major problem in the health care service with a mortality rate of 5% to 10% during the first month and 20% to 30% during the first year after the fracture (Sogaard et al., 2016). After suffering a hip fracture, individuals often have poorer strength, mobility and balance (Madsen et al., 2000; Visser et al., 2000), as well as reduced quality of life (Amarilla-Donoso et al., 2020; Sihvonen et al., 2009) and independence (Langford et al., 2018).

Functional mobility refers to individuals’ ability to move around safely and effectively in their environment; perform basic ambulation; walk for leisure; perform everyday tasks; and participate in work, leisure, and exercise activities (Satariano et al., 2012) and the nature of walking is underlined as an embodied, prereflective and natural act (Martinsen et al., 2018).

A meta-analysis of 13 studies highlighted that exercise and physical activity can be effective in improving the mental wellbeing of individuals aged 65 or older (Windle et al., 2010). Many quantitative studies have investigated the effects of exercise after a hip fracture (Diong et al., 2016; Handoll et al., 2011). However, exercise interventions have proved to have inconsistent effects on mobility (Handoll et al., 2011). A recent meta-analysis reported a minor improvement in overall mobility by following a structured exercise intervention after a hip fracture (Diong et al., 2016). These meta-analyses include studies involving exercise interventions up to 1 year after a fracture (Diong et al., 2016; Handoll et al., 2011). However, the efficacy of exercise interventions in the early recovery phase after a hip fracture has been poorly studied compared with prolonged outpatient interventions (Asplin et al., 2017). Previous qualitative studies on exercise interventions have shown that older adults encounter challenges when recovering from hip fractures (Forsberg et al., 2014; Griffiths et al., 2015; Young et al., 2009).

No studies have been identified that investigate older patients’ own reflections and thoughts on the impacts of evidence-based exercise programs on their mobility in the early recovery phase after suffering a hip fracture. Furthermore, in terms of focusing on strengths and positive resources rather than deficits, information on the factors that might facilitate the recovery process is lacking (Buddingh et al., 2013; de Morton et al., 2007).

By knowing more about what is important for individuals who have experienced a hip fracture, health care services could be better tailored to increase participation and adherence for this particular group. Antonovsky’s notion of *salutogenesis* links health to energy, inspiration, creativity, a sense of coping, self-efficacy, and a secure identity (Gray & Kabadaki, 2005) and will be used to frame our discussion in this article.

### Aim

Our aim with this article was to explore the experiences of older people who had participated in the evidence-based High-Intensity Functional Exercise (HIFE) Program during the first three weeks of rehabilitation after hip fracture surgery.

### Conceptual Framework

According to Bhattacharya et al. (2020), the concept of salutogenesis can contribute to our understanding of the development and maintenance of health. A salutogenic approach, also termed as strengths perspective, emphasizes patients’ unique attributes, talents, abilities, capacities, hopes, values, visions, and knowledge, rather than focusing solely on their problems, difficulties, needs, and deficits (Gray & Kabadaki, 2005). Antonovsky’s salutogenic model for health provides insight into why people—despite stressful situations and hardships—manage to stay well, and represents an alternative to the pathogenic model (Antonovsky, 1979; Antonovsky & Sjøbu, 2012). A central concept in the salutogenic model is the multidimensional health continuum on which people move throughout their lifespans and this model constitutes a rejection of the dichotomous classification of people experiencing either “ease” or “disease” (Antonovsky & Sjøbu, 2012).

The term sense of coherence (SOC) is described as an individual’s ability to comprehend a stressful situation holistically, their capacity to use the available resources, and the extent to which they have a pervasive and enduring, although dynamic, feeling of confidence that the events they experience in life are comprehensible, manageable, and meaningful (Antonovsky & Sjøbu, 2012). Another key concept of the theory is generalized resistance resources (GRRs), which encompass the resources available to make a transition to good health possible and which can be found both within people, bound to their person and capacity, and in their immediate and distant environments (Antonovsky, 1996). Typical GRRs are money, knowledge, experience, self-esteem, healthy behavior, commitment, social support, cultural capital, intelligence, traditions, and view of life (Lindström & Eriksson, 2006).

SOC is therefore considered to be a factor that can elucidate the findings of this study and is worth considering in rehabilitation.

## Method

We employed a qualitative approach and conducted semi-structured interviews to explore participants’ experiences from the exercise sessions (Malterud, 2011). The interviews were obtained when the participants had fulfilled the program of daily one-on-one exercise lessons for 10 days during their stay in a rehabilitation care unit. Our study followed a randomized controlled trial (RCT) format (no. NCT02815254), which involved an evaluation of the HIFE Program in eastern Norway.

### Ethics

Our study and the RCT were approved by the Norwegian Regional Ethics Committee (2015/1814 REK south-east B). All participants were informed about this study both in writing and verbally. Participants gave informed, written consent to participate in the study and to publish the results. The participants were also informed about the possibility of withdrawing their consent to participate at any point in the study without any consequences to their treatment.

### The HIFE Program

The objective of the HIFE Program is to improve participants’ lower limb strength, balance, and mobility (Littbrand et al., 2006; Rosendahl et al., 2006). The HIFE sessions conducted under this study involved 5 minutes of seated warm up, two or more balance exercises, and two or more exercises to strengthen the lower limbs. The instructors used weighted belts, and the exercises were tailored to each individual, based on the participant’s functional level and current health status. Four physiotherapists who had 6 to 13 years of experience in geriatric rehabilitation were involved in the intervention as instructors.

### Participants

Thirteen women and six men with a mean age of 86 years were interviewed. At the time of the interviews, 12 were living alone, whereas seven were living with a spouse, all of them in an urban, central, and high-cost district of Norway. All participants had 2 to 6 years of education above the upper secondary level. Furthermore, all participants used either a pulpit aid or a rollator when they started the program and 10 of the participants had exercised before the hip fracture. Cognitive function was measured on a scale of 0 to 30 by administering the Mini-Mental State Examination (MMSE; Folstein et al., 1975). The scores of our participants were in the range of 17 to 30, with a mean score of 24.6 after the intervention. Prior to the intervention, the mean MMSE score was 23.5. To assess function, we used the Short Physical Performance Battery (SPPB) assessment tool, where the total score range is 0 to 12, and high scores reflect superior function (Guralnik et al., 1994). Our participants’ mean score on this test was 4.3 at baseline and 6.6 on completion of the intervention. Measures of walking distance (meters per minute) had a mean of 106.5 at baseline and 201.5 after the intervention. Health-related quality of life was assessed using a physical and mental health summary from the 36-Item Short Form Health Survey (SF-36), referred to as the PCS-36 and MCS-36, respectively (Ware, 2000). The mean scores of our participants increased during the exercise period from 30.85 to 36.40, according to the PCS-36, whereas the mean score of the MCS-36 before and after the intervention was 46.02 and 46.07, respectively.

### Recruitment and Interviews

The participants were all recruited from the same rehabilitation unit. The inclusion criterion was the fulfillment of the exercise intervention in the RCT, which had the following criteria: having experienced a hip fracture surgery with immediate admission to the rehabilitation unit, aged 65 years or older, home dwelling, able to walk independently indoors with or without a walking aid, and medically found able to provide informed consent to participate. The exclusion criterion for the RCT and for our study was dizziness, which could affect participants’ ability to fulfill the exercise program.

The physiotherapists who instructed the exercise program provided individual information about this qualitative study as the patients arrived at the rehabilitation unit and were enrolled in the RCT. Consent for participation and publication were obtained before the interviews were scheduled. All participants who were asked agreed to be interviewed in this study. When we had completed 18 to 19 interviews, the interviews gave no substantial new information, and the study reached saturation. Malterud et al.’s (2016) model of information power, which considers the specific study aim, sample specificity, use of established theory, dialogue quality, and strategy of analysis, was applied. Malterud et al. (2016) suggest 10 to 25 participants as a sufficient sample size for qualitative studies.

The interviews were performed between October 2017 and May 2018, and a semi-structured interview guide (found as Supplemental File 1) with open-ended questions was used to encourage the participants to talk as freely as possible about their experiences. The interviews lasted approximately 50 minutes, and the participants were informed about the interviewer’s background as a physical therapist and a researcher.

### Analysis

The audiotaped interviews were transcribed in Norwegian by a professional transcriber. We used Malterud’s (2011, 2012) method of systematic text condensation for the analysis, which starts by reading through the transcripts with an open mind for all authors. In the next step, we read through the data again searching for “meaning units” in the text related to the aim of the study. We coded and analyzed the interview transcripts to identify key topics, and this process resulted in a total of 423 codes. In the third step, insight into the meaning units was expressed more directly, as exemplified in Supplemental File 2. In the last step of the analysis, the transformed meaning units were synthesized into statements about the participants’ experiences. The transcripts were translated from Norwegian into English at the end of the analysis process.

Based on the results from the analysis, the patients’ descriptions of their experiences of mobility during the exercise program were grouped into categories under one overarching theme: “Exercise is key to regaining mobility and a sense of coherence (SOC) in everyday life.” This overarching theme summarizes the most salient findings from the data with the following five themes emerging from the analysis: (a) understanding the existential importance of mobility; (b) maintaining a positive self-image by regaining mobility; (c) regaining one’s old life and independence in everyday life; (d) maintaining interpersonal relationships through mobility; and (e) creating positive emotions by being able to move. Examples from the analysis process are found in Supplemental File 2.

### Trustworthiness

Qualitative research involves establishing credibility, transferability, dependability, and confirmability (Lincoln & Guba, 1985). In this study, credibility is attached to the quality of the interviews and the analysis process. Before and when performing the interviews, the interviewer reflected on own background and prejudices to enhance awareness of her preconceptions, thereby supporting the credibility of the study. All authors were actively involved in the analysis and interpretation process, and we attempted to be aware of our preconceived ideas and our influence on the findings, as well as how well the categories represented the data. The challenge of whether the findings can be transferred to other contexts, settings, or groups (Lincoln & Guba, 1985) was addressed by performing an in-depth, detailed, and descriptive analysis of the data and quoting the participants’ responses to substantiate the findings. According to Lincoln and Guba (Lincoln & Guba, 1985), this can provide external validity or transferability. Dependability refers to whether the findings are consistent and stable (Lindseth & Norberg, 2004). The fact that the same researcher conducted all the data collection could help to ensure consistency between and throughout the empirical data in the study. Confirmability refers to the extent to which the findings are shaped by the participants and not by the researchers’ biases, motivations, and interests. Due to our background as health care professionals, we strove to be aware of our preconceptions by discussing the findings with our interdisciplinary research group and actively looking for the unexpected in the data. Nevertheless, we realize that our preconceptions of exercise and physical activity may have influenced our interpretations.

## Results

The overarching theme “Exercise is key to regaining mobility and a sense of coherence (SOC) in everyday life” features the process of change and incorporates the participants’ experiences of how to cope with physical, psychological, and social disabilities. Exercise further contributed to the participants’ perception of the possibility of having a mobile life and provided multidimensional opportunities with broad positive impacts on their everyday lives. The following sections present the themes, underpinned by quotes from the participants.

### Understanding the Existential Importance of Mobility

By experiencing a hip fracture, the participants became aware of their legs as physical objects over which they no longer had control. Through exercise, the participants sought to enhance their walking distance and their capacity by pushing their limits in the face of pain and exhaustion. Being able to walk “normally” without any devices meant a lot for their sense of freedom and happiness, and their ability to fulfill personal needs, as expressed in the following quotation: “To stay on my feet and walk . . . yes. That is an ability I will fight my whole life to keep, and it is necessary for me to retain my courage in life” (female, 70–79 years).

The participants’ realization that their legs were the most important tool to maintain their relationship with their environment by regaining their walking ability became evident in this comment: “My legs are important parts of me; they carry me through life. The activity of walking is the best thing that can happen” (male, 70–79 years).

At the turning point between the incidence of the hip fracture and the start of the exercise program, the participants’ description of their thoughts seemed to be related to existential themes: “When you get older and, at the same time, experience a hip fracture, you are no longer certain of what’s on the other side. I consider this fracture existential. ‘What have I been thrown into now?’ I thought” (female, 80–89 years).

The exercises seem to have helped the participants to regain their existential abilities, in this case being able to use their legs to walk independently.

### Maintaining a Positive Self-Image by Regaining Mobility

The participants appeared to define the word self-image in a broad context, relating it to the meaning of life, mobility, mastering, continuity, possibilities, independence, self-esteem, self-realization, positivity, and control. Their physical decline had consequences for their self-image. As the exercise gradually restored the participants’ sense of coping, their self-image was strengthened. One participant’s description of her thoughts about this concept implied a change during the exercise period in which the instructor’s way of being played an important role:

I felt that my self-image was in danger of becoming really bad when I started out. Strengthening my self-esteem, as I perceived the therapist to do with their instructions, was the factor of success; I think that I must believe that I’m going to make it and that what we are doing matters to me and my situation. The exercise helps me to realize myself, and for this I need energy; it’s about my self-image and how to preserve a positive self-image. I believe that this is fundamental to my motivation for exercise. Perhaps these needs are becoming stronger in my current situation, where I have a broken hip. I also like the experience of a having a strong body—a body that can move me around. I *am* my body, and if my body does not respond, I find my life to be rough. That is why I want to put effort into the exercise. (Female, 70–79 years)

In the following quotation, the meaning of life is portrayed as the connection between the participant’s self-image and the restoration of their ability to move, which underlines the strong feelings brought about by the hip fracture in these participants’ minds:

Being able to continue with the activities I have done before is extremely important. I also find that these activities are central to my feeling of *self-image* and that they add meaning to my life. Exercise is good for the mood and for the body, and it creates the possibility to regain the abilities I had before the fracture. In addition, it gives me a break from negative thoughts. It helps my self-image and gives me the experience of managing and being capable—it gives me back control over my own life. (Female, 90–99 years)

Mobility therefore seemed significant to the participants’ ability to regain and maintain their identity and a positive self-image.

### Regaining One’s Old Life and Independence in Everyday Life

Our participants talked about values such as independence, self-reliance, autonomy, and individualism and underlined the importance of these values in relation to their everyday lives. Exercise was perceived to be meaningful in the process of regaining their ability to manage in their own homes. The need to both be independent and depend on others was reflected in the words of this participant:

I wish to manage my daily life to the extent possible. Even though I am glad to get help when I really need it, I do not want it *until* I need it; otherwise, it could be like an excuse for doing nothing. The maintenance of as much independence as possible is my main motivation for exercising—not to be a burden to other people. (Female, 80–89 years)

The way in which the exercises were organized and integrated with everyday movements was meaningful for the participants because it helped them to understand the utility and transfer value of the exercises in their everyday lives:

The exercises—the number of repetitions, squats, stand up and sit down, step-ups, stairways, walking in different directions, stretching in different directions –were useful and integrated into my daily life. I experienced that independence is threatened in old age, especially after the hip fracture. My therapist thinks that the arrangement will help build up my capacity to manage my life by myself to the extent possible, and that is exactly what I want. (Male, 80–89 years)

### Maintaining Interpersonal Relationships Through Mobility

A mobile body was essential to how the participants experienced the world and their perceived ability to maintain relationships and roles in their lives. Their statements indicated that relationships were at the very core of what made life meaningful for them as older people. The following quotation revealed the importance of social participation, which the participants underlined as essential: “My ability to move says something about my possibility for social participation. It gives me freedom, choice and independence, which are central to my quality of life” (female, 80–89 years).

The role of being a grandmother or grandfather seemed especially important and was mentioned repeatedly by the participants: “I exercise not because of appearance but to have a body that works and that allows me to participate in fun things like visiting friends, playing with my grandchildren, and being an active pensioner” (female, 70–79 years). Playing with grandchildren was one of the several benefits they had experienced from performing their exercises, which were demanding in terms of mobility.

### Creating Positive Emotions by Being Able to Move

Exercise helped the participants to improve their ability to cope with stress, the tension of everyday life, anxiety, and depression. In the following quotation, a participant summed up her experience using the concept of vitality, which strongly links mobility to mental changes:

Exercise makes me aware of life and gives me faith that my life situation can be changed. It is a part of my battle to be present in my life. The game between being alive versus dead is the same as being in motion versus motionless. I do not want to be absent. It is not enough to be physically present. I also want to be mentally present. To me, vitality involves experiencing power, energy, and movement. I experience this in my body. The body is important. (Female, 80–89 years)

Furthermore, the participants made multiple statements describing different types of positive emotions following exercise, including optimism, happiness, and being in a good mood: “Walking makes me happy, so I try to walk as much as possible. I like to move. Being able to move is the entry point for many nice experiences” (female, 80–89 years); “These changes have made me care more about others, and I have become more interested in various opportunities. Moreover, I reward myself with positive thoughts about myself” (male, 70–79 years). The participants recognized that their change of mood was evident in their relationships with the people around them and that it made them more approachable.

## Discussion

In this study, improved mobility was described as a benefit from the exercise intervention and had great importance in terms of improving the participants’ coping with everyday life and enhancing their sense of living a meaningful life. Mobility was further perceived by the participants to be a steppingstone to positive emotions, good relationships, independence, a good self-image, and the ability to keep moving in the challenging and vulnerable situation of reduced physical functioning shortly after suffering a hip fracture. The hip fracture itself was perceived as an existential crisis by the participants. The positive changes brought about by the exercise program represented a turning point in their existential crisis.

To shed light on our findings, we use the salutogenic approach and discuss three main topics: (a) hip fracture as a stressor with existential consequences; (b) exercise as a turning point that created motivation and change; and (c) the connection between mobility and self-image.

### Hip Fracture as a Stressor With Existential Consequences

The hip fracture represented sudden and significant changes in the participants’ lives owing to reduced functional abilities and quality of life. Antonovsky’s (1996) description of stress as a sudden and noncontrollable event covers the descriptions of the hip fracture and its consequences well. Hagsten et al. (2004) found that many older people perceive a hip fracture to be the end of independent life. This underlines the severity and existential nature of this functional loss and aligns with our findings. It has been stated that 50% of patients who experience a hip fracture are expected to need assistant to participate in daily activities (Osnes et al., 2004), while 35% become permanently incapable of walking independently after a hip fracture (Bertram et al., 2011). These numbers indicate that, for many patients, a hip fracture leads to a loss in independence (Langford et al., 2018) caused by reduced mobility. This, in turn, can evoke concern and uncertainty about the future. To older people, independence is ranked as top three of the most important things in life in a study focusing on quality of life (Prieto-Flores et al., 2010). On this background, the participants’ focus on returning to their prefracture functional level and regaining as much independence as possible is understandable. The feeling of being dependent on others might represent that of being a burden. Based on the available evidence, the feeling of dependency and of being a burden might, according to Rodríguez-Prat et al., add to the stressful situation, having a detrimental effect on patients’ quality of life and sense of dignity (Rodríguez-Prat et al., 2019). This can be understood in line with Antonovsky’s (1996) description of human stress, involving psychic, somatic, and social aspects. Our study provides an understanding of the magnitude of the consequences a hip fracture can entail for an individual at a psychic, somatic, and social level, and the intertwined nature of those aspects. The fracture and its physical consequences made participation and socialization difficult for our participants, which greatly affected both their state of mind and quality of life and a movement in the direction of the unhealthy end of Antonovsky’s continuum of health (Antonovsky & Sjøbu, 2012).

### Exercise as a Turning Point That Created Motivation and Change

From experiencing an overload of physical, psychological, and social losses owing to the hip fracture, the participants described the part-taking in the exercise program at an early point in the recovery phase as a turning point, although it was considered demanding in terms of energy. Experiencing the contrast between being hospitalized and in surgery to being able to stand and perform exercises, with or without walking aids, represented a huge step forward in terms of function and a movement toward the healthy end of the health continuum (Antonovsky & Sjøbu, 2012). Doing exercise was a good strategy in the participants’ current situation, and their stories indicated that, by increasing mobility, exercise helped them to improve their experience of wellbeing, despite the stress created by the hip fracture. This is in line with the results of Franco et al.’s (2015) systematic review of studies examining physical activity in patients with different diagnoses, the results of which indicated that 78% of the participants believed that exercise was important for maintaining general health and mood and for relieving stress. Being part of a society where an active and health-promoting lifestyle is highly valued, as well as the fact that the participants were resourceful due to their standard of living and educational level, may have influenced their decisions and positions concerning exercise.

Exercising is considered an aspect of a healthy behavior, which can be a general resistant resource (GRR) in a salutogenic perspective (Lindström & Eriksson, 2006). Having these kinds of resources at your disposal or in the immediate surroundings can be helpful in dealing with challenges of life and constructing coherent life experiences (Lindström & Eriksson, 2006). Exercise as an external intervention can be understood as an environmental resistance resource; however, internal resources, such as motivation, are also necessary to perform the exercises. Motivation is further considered to be strongly connected to the meaningfulness concept in SOC (Antonovsky & Sjøbu, 2012; Langius et al., 1992), and the restoration of mobility was highlighted by the participants as meaningful because it helped them to return to their prefracture functions, and thereby fostered new motivation. This, in turn, appeared to give them what Antonovsky described as a feeling of confidence that life’s events are comprehensible, manageable, and meaningful (Antonovsky & Sjøbu, 2012). This feeling of confidence was linked to the roles as grandparents, where having strength and balance to be able to play with their grandchildren was a necessity and a goal that enhanced their motivation to exercise. Walking together with others, like some of our participants did, was also found by Grant et al. (2017) to be connected to sheer fun and enjoyment of the experience, tied to opportunities for socializing or for fostering new or deepened friendships with other walkers. Antonovsky’s concept of salutogenesis and GRRs link health to positive experiences, such as energy, inspiration, creativity, a sense of coping, self-efficacy, and a secure identity (Antonovsky, 1996), all of which were salient in our findings. Exercise and the regaining of mobility also contributed to the participants’ happiness, which strengthened motivation for further change, as supported by Bourret et al. (2002) and Windle et al. (2010).

### The Connection Between Mobility and Self-Image

The strong position of mobility and its connection to self-image was evident from the participants’ testimonies. The word self-image was used by the participants in connection with self-esteem, self-realization, and other descriptions concerning their identity and important aspects of their existence. This is supported by Moore et al. (2014) who also found a connection between movement and self-identity among older people living with chronic pain, as movement and being physically active were a natural part of their self-identity.

From the salutogenic perspective, persons with a strong identity and a strong feeling of self also have a strong SOC (Antonovsky & Sjøbu, 2012). The “drop” in the participants’ self-image as a result of the hip fracture, and the regaining of it along with the regaining of mobility, influenced the participants’ daily functioning, social participation, relationships with others, and self-image, which underlines the connections between these concepts. It is also concluded that SOC has a unique relation to general health, and the concept also seems to reflect an active self-esteem structure and self-determination (Johnson, 2004).

Mobility played an important role in the participants’ prefracture lives and history, and, as pointed out in Antonovsky’s theory, the strength of a person’s SOC is related to their fundamental history and important life areas (Antonovsky & Sjøbu, 2012). Altogether, the process of change restored some elements of the participants’ lives, providing them with meaning and improving their quality of life.

The ability to walk was described both as a means to an end (i.e., to achieve independence and participation) and as an activity with deep roots in our participants’ histories. Walking seems to be the most existential mobility activity according to our participants, and their testimony was that they would fight to maintain this ability. Langford et al. (2018) also found walking to be the most frequent mentioned kind of activity among a group of older adults after suffering a hip fracture. To understand the existential meaning of walking, we can turn to the individuals’ responses to the loss of this ability—caused in this study by a hip fracture—because the ability to walk is seldom reflected upon until walking difficulties occur (Martinsen et al., 2018). Norlyk et al. (2013) linked the loss of the ability to walk to a limitation of the person’s action space and a loss of freedom (Norlyk et al., 2013), and these losses seem to lie behind our participants’ experiences and might have been reasons for their feelings of alienation and diminished self-image. One female participant expressed that she believed she was her body, and when her body no longer responded as it had before, she found life to be rough. This indicates the connections between body and mind, as well as between mobility and identity.

Mobility in the form of walking is claimed to have a hidden importance (Martinsen et al., 2018), and Bourret et al. (2002) found mobility to be so much more than just movement from one point to another for older people, like our participants who painted mobility as an existential factor.

## Strengths and Limitations

Our findings, as is the case in all qualitative research, are bound to the participants and the study setting (Maxwell, 2012). The participants’ testimonies were especially rich and detailed in content, which may represent a strength in relation to the study’s credibility. The participants’ engagement in the subject area was strong, and most of them had a positive attitude toward exercise, which can be seen as a weakness due to the lack of critical voices. Although all the researchers strove to be aware of their preconceptions and preexisting knowledge (e.g., in the physiotherapy field), the influence of these on the study could represent a threat to its credibility and confirmability. In contrast, their knowledge and experience in the field also made it possible to highlight important topics and questions about the participants’ experience of the exercise program.

We did not include persons with ethnic backgrounds other than Norwegian in this study, and all the participants were residents of an urban part of the country. This may limit the transferability of this study to other cultural settings or to rural districts. A factor that strengthens the transferability of this study, however, is the overrepresentation of women in the sample (13–6) because patients who suffer a hip fracture are predominantly women. Moreover, we conducted this study in a single rehabilitation unit, which may limit the transferability of the findings to other units. Although there are differences between short-term rehabilitation units and the organizational models they employ (Pearson et al., 2015), we believe that other short-term rehabilitation units are comparable to those used in this study because they involve the same mechanisms in terms of purpose, structure, function, and content (Godfrey et al., 2005).

## Conclusion

Our findings support and extend the existing evidence base about the importance of exercise in health care services during the first weeks after hip fracture surgery. Exercise was contextualized and embedded into everyday life as a strategy for regaining mobility, which had an essential position in the participants’ lives. The study further underlines the importance of considering the person’s internal motivation processes when designing and presenting exercise program to maximize the likelihood of implementation and adherence. The present findings may be useful to health care professionals involved in exercise interventions, and especially to those working with vulnerable groups. They may also help decision-makers in the health care services to more effectively develop and tailor health care services for this group of patients.

## Supplemental Material

sj-docx-1-qhr-10.1177_10497323211008848 – Supplemental material for Mobility—A Bridge to Sense of Coherence in Everyday Life: Older Patients’ Experiences of Participation in an Exercise Program During the First 3 Weeks After Hip Fracture Surgery Click here for additional data file. Supplemental material, sj-docx-1-qhr-10.1177_10497323211008848 for Mobility—A Bridge to Sense of Coherence in Everyday Life: Older Patients’ Experiences of Participation in an Exercise Program During the First 3 Weeks After Hip Fracture Surgery by Irene Vestøl, Jonas Debesay and Astrid Bergland in Qualitative Health Research

sj-docx-2-qhr-10.1177_10497323211008848 – Supplemental material for Mobility—A Bridge to Sense of Coherence in Everyday Life: Older Patients’ Experiences of Participation in an Exercise Program During the First 3 Weeks After Hip Fracture Surgery Click here for additional data file. Supplemental material, sj-docx-2-qhr-10.1177_10497323211008848 for Mobility—A Bridge to Sense of Coherence in Everyday Life: Older Patients’ Experiences of Participation in an Exercise Program During the First 3 Weeks After Hip Fracture Surgery by Irene Vestøl, Jonas Debesay and Astrid Bergland in Qualitative Health Research
