# Supplementary material for: Mobility—A Bridge to Sense of Coherence in Everyday Life: Older Patients’ Experiences of Participation in an Exercise Program During the First 3 Weeks After Hip Fracture Surgery
Source: Qual Health Res. 2021 Apr 30;31(10):1823–32. doi: 10.1177/10497323211008848 (PMC8446889; doi:10.1177/10497323211008848)
Supplement: sj-docx-1-qhr-10.1177_10497323211008848 – Supplemental material for Mobility—A Bridge to Sense of Coherence in Everyday Life: Older Patients’ Experiences of Participation in an Exercise Program During the First 3 Weeks After Hip Fracture Surgery [file sj-docx-1-qhr-10.1177_10497323211008848.docx]

**Supplementary file 2.**

Examples of the analysis process

Overarching theme: Exercise is the key for regaining mobility and a sense of coherence (SOC) in everyday life

| **Participant** | **Meaning units** | **Codes** | **Condensation** | **Theme** |
| --- | --- | --- | --- | --- |
| Female,  70–79 | “To stay on my feet and walk . . . yes. That is an ability I will fight my whole life to keep, and it is necessary for me to retain my courage in life.” | - To walk is a dream. - The ability to walk is worth fighting for. - Walking is necessary to retain courage in life. | Being able to walk and have control over my body means everything to my motivation and gives me a feeling of freedom and courage. | Understanding the existential importance of mobility |
| Male,  80–89 | “Yes, you see, I find that having control over my body means everything to me. To be able to go outdoors and move is a motivation.” | - Body control is important. - The freedom of walking outdoors is a motivation. | Being in control over my body and be able to move outdoors means everything and gives me a feeling of freedom and motivation. |  |
| Male,  70–79 | “My legs are important parts of me; they carry me through life. The activity of walking is the best thing that can happen.” | - Legs are important body parts. - *Legs carry me through life.* - Walking is the best activity. | My legs are important parts of me, and they carry me through my life. The hip fracture was an existential event that made me uncertain about my future. |  |
