# Supplementary material for: Mobility—A Bridge to Sense of Coherence in Everyday Life: Older Patients’ Experiences of Participation in an Exercise Program During the First 3 Weeks After Hip Fracture Surgery
Source: Qual Health Res. 2021 Apr 30;31(10):1823–32. doi: 10.1177/10497323211008848 (PMC8446889; doi:10.1177/10497323211008848)
Supplement: sj-docx-2-qhr-10.1177_10497323211008848 – Supplemental material for Mobility—A Bridge to Sense of Coherence in Everyday Life: Older Patients’ Experiences of Participation in an Exercise Program During the First 3 Weeks After Hip Fracture Surgery [file sj-docx-2-qhr-10.1177_10497323211008848.docx]

**Supplementary file 1**

**Semi-structured interview guide**

Can you please describe how you fell and fractured your hip and what this meant to you?

Can you please describe how this influenced what you do in your everyday life?

Can you please describe you experience of participating in the exercise program?

Can you please describe what is most important for your motivation to exercise?

Can you please describe what was most helpful about the exercise program?

Can you please describe if you have noticed any changes that may be a result of doing exercise, and if you have, how do you experience them?

Can you please describe if/how your activity levels change after the exercise program had finished?

Can you please describe your experience of the instruction of the exercises?

Can you please describe if/how you set goals for the exercise? (alone or together with the exercise instructor/physiotherapist?)

Can you please describe if you met your goals?

Can you please describe if/how important other people are in influencing your recovery?
